# Couples' Joint Health Behaviors Predict Better Health and Stronger Resemblance Between Partners

**DOI:** 10.1093/geroni/igaa057.1944

**Published:** 2020-12-16

**Authors:** Stephanie Wilson, Joshua Novak

**Affiliations:** 1 Southern Methodist University, Dallas, Texas, United States; 2 Auburn University, Auburn, Alabama, United States

## Abstract

Satisfying marriages pose benefits and possible risks to health. Indeed, high-quality relationships boost emotional resources and encourage healthy behaviors. However, stress and its adverse health effects also spread more easily in close relationships. To examine the relevance of joint health behaviors for health indicators and partners’ health similarity, 227 couples age 23-84 reported their frequency of co-sleeping, exercising together, and sharing meals; relationship satisfaction; health satisfaction; recent medical visits; and health problems. Happier couples shared more joint health behaviors than unhappier counterparts. In turn, joint health behaviors predicted greater health satisfaction and more similar rates of health problems between partners. In particular, exercising together predicted greater health satisfaction, fewer health problems, and greater health similarity. Controlling for diet, sedentariness, and sleep, findings revealed that joint health behaviors—a characteristic of happy relationships—are linked to not only better health and greater health satisfaction, but also greater health similarity between partners.

